# Principal Feature Analysis: A Multivariate Feature Selection Method for fMRI Data

**DOI:** 10.1155/2013/645921

**Published:** 2013-09-21

**Authors:** Lijun Wang, Yu Lei, Ying Zeng, Li Tong, Bin Yan

**Affiliations:** China National Digital Switching System Engineering and Technological Research Center, Zhengzhou 450002, China

## Abstract

Brain decoding with functional magnetic resonance imaging (fMRI) requires analysis of complex, multivariate data. Multivoxel pattern analysis (MVPA) has been widely used in recent years. MVPA treats the activation of multiple voxels from fMRI data as a pattern and decodes brain states using pattern classification methods. Feature selection is a critical procedure of MVPA because it decides which features will be included in the classification analysis of fMRI data, thereby improving the performance of the classifier. Features can be selected by limiting the analysis to specific anatomical regions or by computing univariate (voxel-wise) or multivariate statistics. However, these methods either discard some informative features or select features with redundant information. This paper introduces the principal feature analysis as a novel multivariate feature selection method for fMRI data processing. This multivariate approach aims to remove features with redundant information, thereby selecting fewer features, while retaining the most information.

## 1. Introduction

Over the past decade, multivoxel pattern analysis (MVPA) has become widely used in functional magnetic resonance imaging (fMRI) because of its effectiveness in decoding cognitive states [1–7]. Unlike univariate statistical methods focusing on characterizing the relationship between cognitive variables and individual brain voxels, MVPA applies powerful pattern classification algorithms to multivoxel patterns of activity to decode the information that is represented in that pattern of activity [1]. In general, MVPA considers each multivoxel pattern (referred as a sample) as an *n*-dimensional vector and separates these vectors in the high-dimensional space by classification [8]. Multivoxel patterns are first divided into training and test sets, after which a classifier is trained on the training set and tested on the test set. A cross-validation procedure is required to accurately estimate the performance of the pattern classifier. This procedure is implemented by dividing the data of all samples into portions of equal size. At each cross-validation, one portion is left out, the classifier is trained on the remaining portions, and predictions are made for the portion that was left out. The mean accuracy of all cross-validations serves as the final estimate of the classifier's true performance.

In MVPA-based analysis, feature selection (i.e., voxel selection) is critical in conducting efficient classifications because the number of voxels corresponding to the feature dimension in MVPA collected from the whole brain is usually very large (up to thousands or more) [9]. Even if the region of interest (ROI) is defined, the number of voxels inside a single region can be up to a thousand. A large ratio of the number of voxels to the number of samples results in overfitting [10]. The generalization capability of the classifier might be reduced if some uninformative voxels are included during the training stage. Feature selection can simultaneously remove uninformative features before pattern classification and reduce the number of dimensions in the multivariate space in which the classifier operates, thus alleviating the danger of overfitting.

The main motivation for using feature selection is to improve the classification accuracy by reducing the size of the feature set. The goal is to find a subset of features that leads to the optimum classification performance [11]. Feature selection can be performed by defining a small ROI by anatomical location or a functional localizer [12]. However, feature selection is usually applied by calculating univariate or multivariate statistics [13, 14]. These methods search the space of all possible subsets of features to find the informative ones by their univariate or multivariate statistics. Feature selection methods can be categorized into three broad categories according to search heuristics: scoring/filtering methods, wrapper methods, and embedded methods [11, 15]. Scoring/filtering methods use intrinsic properties of data to rank the features. The features are all scored and then sorted by their scores, and only those with high scores are reserved and then used as an input to the classifier. Most scoring/filtering methods are univariate, wherein each feature is scored by itself. An example is the commonly used *t*-statistics method, which uses univariate *t*-value to rank features. Wrapper methods select new features based on the impact they have on the classifier given the features already selected [15]. In wrapper methods, features are selected in interaction with the classifier, only features that can increase the classification accuracy will be selected. Embedded methods use the parameters of the classifier directly rather than using the classification accuracy to rank features. A typical example is the support vector machine (SVM)-based feature selection method [16]. Embedded methods require less computation than wrapper methods. 

Feature selection methods can also be divided into two classes: univariate and multivariate. Univariate methods use univariate statistics to rank features. For example, features that best discriminate between the conditions of interest individually (i.e., univariate wrapper method) can be selected. Any univariate statistic used in conventional fMRI analysis can be used to score the features. However, univariate methods unavoidably discard features that, when taken in aggregate, would have provided useful information about the experimental conditions [1]. By contrast, multivariate feature selection methods can avoid this problem by computing multivariate statistics for feature ranking because they consider the dependencies between the features when calculating scores for features. As a typical example, searchlight is the most intuitively appealing multivariate voxel selection method; it creates a spherical multivariate searchlight and moves the searchlight through the measured volume. The spherical searchlight is centered on each voxel of the volume in turn during the movement. To combine the signals from all voxels falling into the searchlight, a multivariate effect statistic is computed at each location. Two approaches can be used to perform searchlight. One approach that is easy to implement is to first perform a conventional linear-regression contrast analysis to obtain a *t*-value for each voxel and then average the absolute *t*-values within the searchlight to measure the difference between patterns [14]. Another approach takes the covariance structure of the noise into account by computing the Mahalanobis distance (for more details, see [14]). As an information-based multivariate feature selection method that aims to pick features containing information about the experimental condition, searchlight is sensitive to features that might be discarded by univariate methods.

Feature selection methods rank features by their scores and then select a predefined number of highest scoring features as an input to classifiers. Therefore, univariate and multivariate statistics that can provide a ranking for features can also be used to select features. Principal component analysis (PCA) has long been used to reduce feature dimension [15]; however, PCA is often used as a feature extraction method rather than a feature selection method. In contrast to feature selection methods, feature extraction methods calculate a weighted projection of multiple features onto new dimensions and select a predefined number of dimensions [9]. Classification is performed on these dimensions rather than on the original features. The features transformed by the principal components are not directly connected to the physical nature of original features, thereby complicating the interpretability of the classification. It is not difficult to notice that each eigenvector (i.e., transform coefficients of each principal component) provides a ranking for all features in original space, implying that we can use some eigenvectors to select features. In 2004, Malhi and Gao [17] proposed a feature selection method based on PCA, using the eigenvector corresponding to the eigenvalue with the largest magnitude to rank original features and to choose the most sensitive features from the original feature set. The PCA-based feature selection method provides an accurate classification for machine defect. In 2007, Lu et al. [18] proposed the principal feature analysis (PFA), a novel method of feature selection. In PFA, a subset of original features that contains most of the essential information is selected using the same criteria as PCA. PFA has been successfully applied in selecting principal features in face tracking and content-based image retrieval problems [18].

In this study, we introduced the PFA approach into fMRI data processing and applied it to a four-category (car, face, building, and animal) object classification analysis. The commonly used feature selection methods based on (univariate) *t*-statistics and (multivariate) searchlight were also applied. Combined with SVM classifier, which has been proven efficient in handling high dimensional data, we compared the prediction accuracies of these three methods. The results show that PFA is an effective feature selection method for fMRI data processing because it can retain most of the information with fewer features.

## 2. Materials and Methods

### 2.1. Subjects

Ten healthy subjects (all college students, four females and six males) participated in this fMRI study. The study was approved by the Institutional Review Board of China National Digital Switching System Engineering and Technology Research Center. All participants had normal vision, received information about fMRI, and gave informed consent.

### 2.2. Stimuli

The stimuli consisted of four categories (animal, building, car, and human face) of color images, with 50 different images in each category. All images were cropped to the center (700 pixels × 700 pixels) and placed onto a gray-scale background.

Visual stimuli were rear-projected onto a screen in the scanner bore using a luminance-calibrated LCD projector driven by a PC. The subjects viewed the screen from a mirror. The display resolution was 1024 × 768, and the stimulus presentation script was written using MATLAB (The Mathworks) and Psychtoolbox 3.0 (http://psychtoolbox.org/).

### 2.3. Experiment Design

Each subject participated in three task runs, four localizer runs, and one retinotopic mapping run. The task runs were designed in an event-related fashion, wherein all images were presented in a 4 s stimulus trial. In each trial, an image was first presented for 2 s, and the gray background was presented for the last 2 s. Each presentation consisted of an image being periodically flashed ON-OFF, where ON corresponds to the presentation of the image for 200 ms, and OFF corresponds to the presentation of the gray background for 200 ms. The first two task runs consisted of 70 distinct images randomly presented once each time. The last task run consisted of 60 distinct images also randomly presented once each time. A blank trial that lasted for 4 s was conducted as a break after every five stimulus trials. The task runs were used to perform classifications.

In the localizer runs, the subjects were presented with blocks of images for each category. Data acquired from this part was used for feature selection. Different datasets were used to avoid the dangers of double dipping (for more details, see [19]). Each run consisted of 12 blocks, with 6 task blocks and 6 control blocks. The task blocks lasted the same time as the control blocks (30 s). Each localizer run consisted of six images randomly selected from the same image category. Each task block consisted of an image being periodically flashed ON-OFF, where ON corresponds to the presentation of the image for 200 ms, and OFF corresponds to the presentation of the gray background for 200 ms. These four localizer runs were used to perform feature selections. The object responsive (OR) voxels were a set of voxels that were strongly activated in at least one localizer run (a spherical searchlight with a two-voxel radius, comprising 33 voxels, average absolute *t*-value, 3,000 voxels selected for each localizer run, and approximately 4,300 voxels selected in total).

Standard retinotopic mapping run with polar stimuli was performed to delineate the early visual areas on a flattened cortex. The visual voxels were a set of voxels located in the early visual areas (approximately 1,290 voxels).

### 2.4. Data Acquisition

All fMRI data were acquired on a 3-T GE Discovery MR750 scanner (General Electric, Fairfield, Connecticut, USA) with a standard head coil at the Imaging Center of Henan Province. For each participant, a standard gradient-echoplanar imaging series was used to collect functional images with the following parameters: repetition time (TR), 2000 ms; echo time (TE), 30 ms; field of view (FOV), 220 mm × 220 mm; matrix size, 64 × 64; 39 slices; slice thickness, 3.5 mm; flip angle (FA), 80°; acquisition voxel size, 3.44 mm × 3.44 mm × 3.5 mm. In addition, a high resolution, three-dimensional T1-weighted structural image was acquired with the following parameters: TR, 8.268 ms; TE, 3.24 ms; FA, 12°; matrix size, 256 × 256.

### 2.5. Data Preprocessing

Functional brain volumes were preprocessed with SPM8 (Statistical Parametric Mapping, http://www.fil.ion.ucl.ac.uk/spm/software/spm8/) and REST (http://www.restfmri.net/). The first 10 volumes of each run were discarded because of the instability of the initial MRI signal and the adaptation of subjects to the circumstance. Slice timing was performed on all functional images. The images were realigned to the first image in the first run for motion correction. We used REST to remove the linear drift in each run.

For retinotopic mapping analysis, FreeSurfer (http://surfer.nmr.mgh.harvard.edu/) was used to reconstruct a T1-weighted anatomical image. The realigned retinotopic mapping images were registered to the anatomical image to obtain the registration file. The following retinotopic analysis was consistent with the one performed in [20].

### 2.6. Voxel Selection Using PFA

#### 2.6.1. Background and Notation

Suppose matrix  **Y**  is the fMRI data with the dimension  *n* × *L*,

(1) $$\begin{array}{c} Y=\left(y_{1},y_{2},\ldots ,y_{L}\right),\;y_{i}\in R^{n}, \end{array}$$

where  *n*  is the number of voxels, and  *L* is the number of observations. Let  Σ  be the covariance matrix of  **Y**  and  Λ  be a diagonal matrix whose diagonal elements are the eigenvalues of Σ,  Λ = diag⁡(*λ*_1_, *λ*_2_,…, *λ*_*n*_), *λ*_*i*_ ≥ *λ*_*i*+1_. Then,  Σ  can be written as

(2) $$\begin{array}{c} \Sigma =A\Lambda A^{T}, \end{array}$$

where  **A**  is the eigenvector of the covariance matrix Σ, written as **A** = (**e**_1_, **e**_2_,…, **e**_*n*_),  **e**_*i*_ ∈ **R** ^*n*^, and  **e**_*i*_  is an eigenvector corresponding to the eigenvalue  *λ*_*i*_. Let  **A**_*q*_  be the first  *q*  eigenvectors and  **a**_*i*_  be the row vectors of  **A**_*q*_:

(3) $$\begin{array}{c} A_{q}=\left(e_{1},e_{2},\ldots ,e_{q}\right)={\left(a_{1},a_{2},\ldots ,a_{n}\right)}^{T},\;a_{i}\in R^{q}, \end{array}$$

where each vector  **a**_*i*_  represents the projection of the *i*th feature of  **Y**  to the *q*-dimension space. Features that are highly correlated or have high mutual information will have similar weight vectors  **a**_*i*_, which can be used to remove features with redundant information. Based on this, PFA finds the highly correlated features and removes those with redundant information.

#### 2.6.2. PFA Algorithm

PCA is first applied on  **Y**, after which we get all the eigenvalues and eigenvectors of the covariance matrix Σ. The first  *q*  eigenvectors are selected to construct the matrix  **A**_*q*_, and the *k*-means algorithm is applied on  **A**_*q*_  to cluster the row vectors  **a**_*i*_  to *k* clusters or more.

Suppose that  ***μ***_1_, ***μ***_2_,…, ***μ***_*k*_  are the centers of *k* clusters, *k* samples are randomly selected to be the initial centers of *k* clusters. All the samples (**a**_1_, **a**_2_,…, **a**_*n*_) are classified according to their cosine distance to the centers  ***μ***_*i*_  (*i* = 1,2,…, *k*)  into *k* classes, that is, *k* clusters. The centers of *k* clusters are recalculated, all samples are reclassified until the centers do not change, and then the final centers  ***μ***_1_, ***μ***_2_,…, ***μ***_*k*_  are obtained. For each cluster, only the vector closest to the center of cluster is retained, and the feature corresponding to this vector is finally selected as the informative feature.

PFA exploits the temporal characteristics and the spatial information for the purpose of feature selection. The temporal information is merged into spatial characteristics when the covariance matrix (or correlation matrix) representing the dependencies between features is calculated. Insights into the original features can be obtained by exploiting spatiotemporal characteristics. Thus, features are selected without redundancy of information.

However, the original PFA method does not take the potentially noisy features into consideration. Noisy features, if existent, will also be clustered to provide at least one feature added to the final feature set. As a result, the classification performance may be reduced. In this study, we use the eigenvector corresponding to the eigenvalue with the largest magnitude to rank the selected features and remove features with small scores.

## 3. Results and Discussion

We combined feature selection methods with SVM classifier to compare the prediction accuracies in the classification analysis. The accuracies were all estimated through 5-fold leave-one-out cross-validation. Generally, we used the voxels as features to study the performance of different feature selection methods (PFA, *t*-statistics, and searchlight). Each feature selection method was applied to the fMRI data to find a subset of preselected voxel sets for each stimulus category. The union of all subsets served as the final subset of voxels, which will be used for the classification. We applied these feature methods to two preselected voxel sets. One is a set of the OR voxels that are strongly activated in at least one localizer run (a spherical searchlight of two-voxel radius, comprising 33 voxels, average absolute *t*-value, 3,000 voxels selected for each localizer run); the other is a set of voxels in early visual areas (delineated on a flattened cortex generated by FreeSurfer software).

### 3.1. Classification Performances of Different Feature Selection Methods

**Figure 1:**
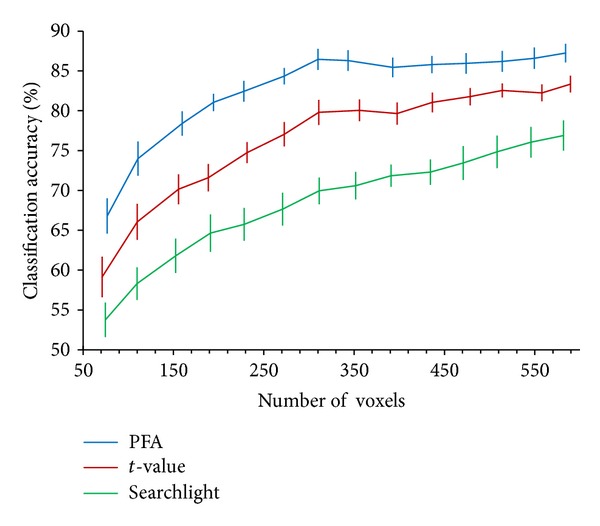
Classification accuracies against the number of voxels for different feature selection methods. PFA: mean classification accuracy of PFA approach across 10 subjects; *t*-value: mean classification accuracy of *t*-statistics approach across 10 subjects; searchlight: mean classification accuracy of searchlight approach (spherical, two-voxel radius, comprising 33 voxels, and average absolute *t*-value) across 10 subjects. Error bars show the standard error of the mean accuracies across 10 subjects.

We compared the mean accuracies of the three methods with different numbers of voxels (Figure 1). As shown in the figure, the accuracies of all three methods increase with increasing voxel number when the number of voxels is less than 310. When the number of voxels is more than 310, the accuracy of PFA does not increase further, whereas the accuracies of the other two methods still increase, especially searchlight. However, the performance is not changed. The accuracy of PFA is still the highest. Compared with the other two methods, PFA retains most of the information about the experimental conditions with fewer voxels.

**Figure 2:**
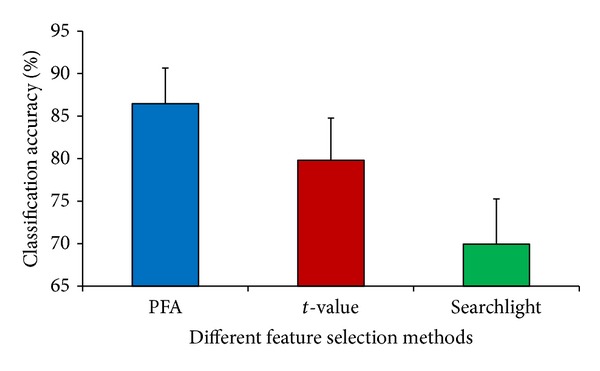
Classification accuracies of different voxel selection methods. For each subject, the feature selection methods were applied to select approximately 310 voxels and to calculate the classification accuracies. Then, mean accuracy was computed across all subjects for each method. Error bars show the standard deviation of the mean accuracies across 10 subjects.

The optimum number of voxels for PFA is approximately 310; thus, we compared the performances of classification with 310 voxels for all methods. For each feature selection approach, we calculated the classification accuracy for all subjects. The mean accuracies across 10 subjects are shown in Figure 2. The average prediction accuracies of the PFA approach are significantly higher than those of the method based on *t*-statistics at the significance level of 0.005 (Wilcoxon signed-rank test). The average prediction accuracies of the *t*-statistics approach are significantly higher than those of the searchlight approach at the significance level of 0.005. PFA outperforms *t*-statistics and searchlight, having the highest classification accuracy. Surprisingly, the multivariate searchlight method does not outperform the univariate *t*-statistics approach.

We have studied the computation cost of the three feature selection methods. To rank and select voxels from OR voxels (with approximately 4,300 voxels), *t*-statistics costs about 54 s and searchlight costs about 55 s (using SPM8). Under the same conditions, PFA only costs 27 s to rank and select voxels. Hence, when focusing on specific brain areas, PFA is still a computationally efficient method compared with the other two methods.

**Figure 3:**
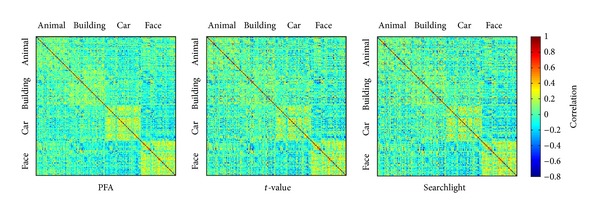
Similarity matrices for stimuli of four categories. The correlations between each pair of patterns corresponding to 200 stimuli (50 stimuli for each category) were calculated. These patterns were normalized to a mean of zero in each voxel across categories by subtracting the mean response across all patterns. The color reflects the values of the correlations (see color bar), patterns with high positive correlations would be classified to the same category, whereas those with low negative correlations would be classified to different categories.

We also applied correlational multivariate analyses to spatial patterns selected by the three methods to compare the sensitivity to differences between the four conditions and have an intuitive understanding on the classification performances. However, some changes were made to demonstrate the within-category and between-category correlations. For each method, we selected 310 voxels from the early visual areas of a single subject S1. The correlations were then calculated between each pair of 200 patterns corresponding to 200 stimuli (50 stimuli for each condition). The results are shown in Figure 3. Comparing the within-category and between-category correlations, the contrast of PFA is slightly higher than contrasts of the other two methods. Hence, PFA outperforms the other two methods in the classification.

As mentioned previously, informative features may be discarded in feature selection when using univariate methods. However, this problem can be avoided with multivariate methods. The classification accuracies of the univariate *t*-statistics approach shown in Figures 1 and 2 suggest that the use of *t*-statistics in feature selection discards voxels that can provide useful information for pattern classification. By contrast, PFA avoids the risk of discarding informative voxels by considering the dependencies of features. When PCA is applied, PFA takes advantage of the correlations of features and then uses the eigenvectors of the covariance matrix, which contains the dependencies of features, to select informative features. Hence, PFA retains most of the information with fewer voxels and has a better classification performance.

Although searchlight is a multivariate method, it has worse classification performance than the univariate *t*-statistics method. In this study, we used a spherical searchlight with two-voxel radius (comprising 33 voxels, average absolute *t*-value). The averaging of absolute *t*-value considers the dependencies of features in the spherical area. Thus, voxels around strongly activated voxels would have high scores, although they may be weakly activated themselves. Searchlight can detect weakly activated voxels; thus, it is more sensitive to focally distributed effects than the univariate activation-based *t*-statistics method [14]. Generally, searchlight can find more activated voxels than *t*-statistics. However, problems arise when only a few voxels are selected to perform classification. Isolated activated voxels may become more weakly activated after conducting searchlight and will be discarded if only a few voxels can be retained. Obtaining high classification accuracies with searchlight is difficult without these informative voxels. Actually, high classification accuracies can also be obtained with searchlight by selecting more voxels (up to a thousand or more).

### 3.2. Distribution of Voxels Selected by Different Feature Selection Methods

**Figure 4:**
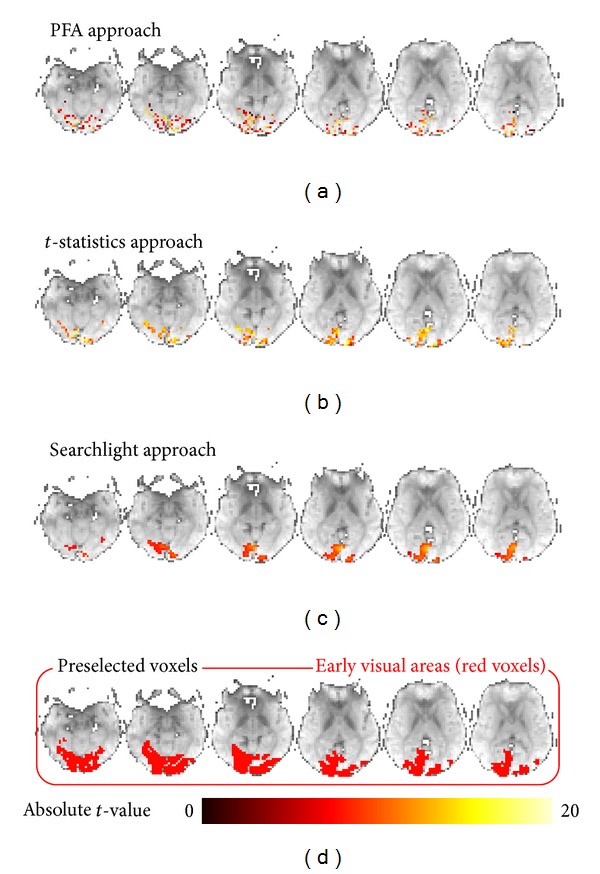
Distributions of voxels (with their absolute *t*-values) selected by different feature selection methods and location of the visual areas of a single subject (S1). For each method, 310 voxels were selected and overlaid on the functional maps. (a) to (c) show the distribution of voxels selected by PFA, *t*-statistics and searchlight (two-voxel radius, comprising 33 voxels, average absolute *t*-value). The color linearly reflects the absolute *t*-value of the voxels shown in (a) to (c) (see color bar on bottom). The visual areas are illustrated in (d). The slices shown in (a) to (d) are slices 12, 13, 14, 15, 16, and 17 of the 39 axial slices acquired. Visual areas are mostly on these slices.

We studied the distribution of voxels selected by the three methods to gain better insights into the final voxel sets. For each method, we selected 310 voxels from visual areas of the subject S1 and overlaid them on functional maps (Figure 4). The color reflects the absolute *t*-values of voxels processed by SPM8. The voxels selected by *t*-statistics mostly have the highest absolute *t*-values, while voxels selected by searchlight mostly have relatively lower *t*-values. However, voxels selected by PFA have a wide range of *t*-values, ranging from almost the lowest to the highest *t*-values. It was concluded that not only the location of large responses carries category-related information, small responses are also an integral part of the representation [12]. PFA did not only select voxels with large responses but also selected those with small responses, thus retaining more category-related information.

As illustrated in Figure 4, the voxels selected by PFA are more distributed and have a wide range of absolute *t*-values. Undoubtedly, voxels with strong activations often contain category-related information. However, some weakly activated voxels are also an integral part of the representation of objects. Activation-based feature selection methods (e.g., *t*-statistics) only select the most activated voxels. Weakly activated voxels are discarded as well as the noisy voxels. Searchlight, as a multivariate method, can detect weakly activated voxels by selecting a larger number of voxels. However, searchlight cannot detect some of these voxels when the number of voxels is limited to a few hundreds. PFA combines the temporal and spatial information to cluster the features; thus, voxels that contain similar information are gathered in the same cluster. Selecting one feature from each cluster to construct the subset of selected features will retain most of the information because different clusters contain different information. Thus, weakly activated voxels gathered in some clusters and have their own opportunity to provide information on the experimental conditions. The most activated voxels are also gathered in several clusters and only several voxels will be selected, thereby removing many voxels containing redundant information. The final subset of voxels becomes more distributed because voxels containing redundant information are removed. Information in fMRI data appears to be presented in a scattered manner [21]. Hence, the distribution of voxels selected by PFA is consistent with the distributed nature of activities, implying that PFA is well suited for selecting distributed voxels with useful information.

### 3.3. Classification Performances of Different Masks

**Figure 5:**
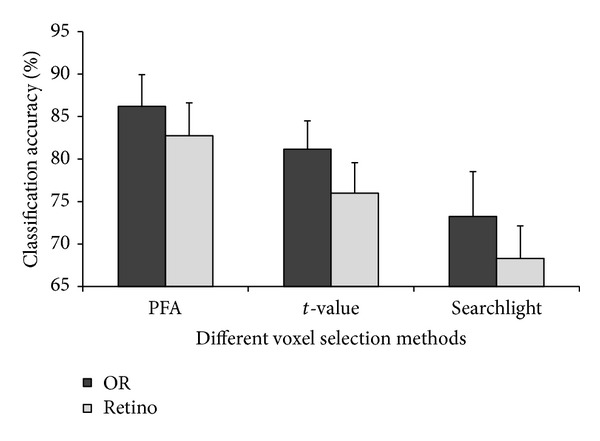
Classification accuracies in OR and early visual areas. OR voxels were strongly activated in localizer runs. Retino voxels were located in the early visual areas, which was delineated via retinotopic mapping analysis. Error bars show the standard deviation of the averaged classification accuracy across 10 subjects.

For each subject, we defined a mask of OR voxels in the occipital cortex that responded strongly in at least one of the four localizer runs. Then, approximately 4,300 voxels were selected for each subject. The voxels in this area were previously shown to provide information about object category [12]. We also tested the classification accuracy in the early visual areas, which were delineated by retinotopic mapping [20]. Three feature selection approaches (PFA, *t*-statistics and searchlight) were applied on OR and visual voxels, respectively. For each method, we selected 310 voxels and calculated the classification accuracy for all subjects. The mean classification accuracies across 10 subjects in both masks are summarized in Figure 5.

These results demonstrated that category-related information has a distributed presentation in the occipital area, not limited in the early visual area. The information contained in occipital area can improve the classification performance. Hence, localizer runs are necessary in object-related brain decoding experiments. Furthermore, in a brain-computer interface (BCI) system with visual information, the spatially distributed information in large areas should not be ignored to obtain a better result [22].

## 4. Conclusion

This study is the first to use PFA for feature selection in brain decoding. PFA is a multivariate feature selection method that exploits spatiotemporal characteristics to select principal features. This method aims to retain most of the useful information with fewer features. PFA uses all the important principal components to exploit the structure of original features. The performance of PFA was evaluated in the four-category classification and correlational multivariate analyses. The results demonstrate that PFA is sensitive to weakly activated features which cannot be detected by the traditional *t*-statistics approach. PFA has a significant improvement on the classification accuracy for visual decoding compared with *t*-statistics and searchlight. In the future, we aim to optimize the cluster algorithm in the component space to achieve better classification accuracy and less computation cost.
